# Male sexual dysfunction in patients with chronic kidney disease: a cross-sectional study

**DOI:** 10.1038/s41598-024-59844-4

**Published:** 2024-04-22

**Authors:** Ruijie Fu, Peihua He, Weihong Hong, Yichun Liang, Wen Wang, Shaoying Yuan, Lichang Liu

**Affiliations:** 1 The Second Clinical Medical College, Guangzhou University of Chinese Medicine, Guangzhou, 510006 China; 2 Department of Nephrology, Zhuhai Hospital of Guangdong Provincial Hospital of Chinese Medicine, Zhuhai, 519015 China; 3 Department of Andrology, Zhuhai Hospital of Guangdong Provincial Hospital of Chinese Medicine, Zhuhai, 519015 China

**Keywords:** Chronic kidney disease, Sexual dysfunction, Erectile dysfunction, Premature ejaculation, Urology, Reproductive disorders

## Abstract

Sexual dysfunction is common in males with chronic kidney disease (CKD), but yet the prevalence and specific relationship between CKD and sexual dysfunction, especially premature ejaculation (PE), remain to be investigated in China; This study aims to examine the prevalence and association between CKD and sexual dysfunction in male patients in China; In this cross-sectional, non-interventional, observational study conducted at a single center. 72 male patients with CKD were enrolled. Data collection included socio-demographic information, assessments via the 5-item version of the International Index of Erectile Function (IIEF-5), the Chinese version of the Premature Ejaculation Diagnostic Tool, the Patient Health Quentionnnaire-9 and the General Anxiety Disorder-7. Data analysis was performed using R version 3.5.2 and SPSS software version 25.0; Among the 72 CKD patients, 56.9% experienced erectile dysfunction and 29.2% had PE. Various factors including estimated Glomerular Filtration Rate, Albumin-to-Creatinine Ratio, psychological aspects, medication use were found to be associated with sexual dysfunction in these CKD patients; Sexual dysfunction is prevalent in males with CKD and is, influenced by multiple factors. It is important for clinicians to focus on sexual dysfunction in this patient group and further investigate its underlying mechanisms.

## Introduction

Chronic kidney disease (CKD) represents a persistent, progressive deterioration of kidney structure and function due to a variety of causes^1^. Globally, CKD prevalence ranges from 10.6 to 13.4%^2^. with recent epidemiological studies indicating that in China, CKD prevalence is around 8.2%, with males comprising 55.4% of this group^3^.

Patients with CKD often exhibit multi-system lesions, with sexual dysfunction being notably associated, as reported in prior research^4^. This dysfunction encompasses a spectrum of disorders, including erectile dysfunction (ED), ejaculatory dysfunction, orgasmic dysfunction, and hypoactive sexual desire disorder (HSDD), with ED being the most prevalent, followed by premature ejaculation (PE)^5^. Notably, a higher incidence of ED has been observed in studies focusing on male CKD patients, particularly those with end-stage renal disease^6^. Research by Guven et al., which included 90 male CKD patients demonstrated that PE scores were significantly higher in this group compared to healthy individuals^7^.

Sexual dysfunction significantly impacts personal quality of life, affecting self-image, self-confidence and self-esteem^8^. Despite its prevalence, sexual dysfunction often remains unrecognized, with only 22% of affected individuals seeking medical assistance^9^. Furthermore, the medical community frequently overlooks this condition, while prioritizing other critical health issues, a situation that is compounded by a lack of clinical experience in addressing male sexual problems. Consequently, recognizing and addressing sexual dysfunction in men is crucial for enhancing a patient’s quality of life.

Nevertheless, there are few international and Chinese studies on sexual dysfunction among CKD patients. Therefore, this study amied to explore the prevalence and associated factors of sexual dysfunction in non-dialysis male CKD patients across various stages by conducting a cross-sectional study in China.

## Materials and methods

### Study design and participants

This study was a cross-sectional, non-interventional, observational real-world analysis conducted at the nephrology and andrology department of Zhuhai Hospital of Guangdong Provincial Hospital of Chinese Medicine, from February to July 2023. Seventy-two male CKD patients wre included based on the following criteria: aged between 18 and 60 years; confirmed CKD diagnosis; and signed informed consent. Exclusion criteria encompassed: absence of sexual activity; diagnosed urogenital cancer or severe conditions like heart failure or psychiatric disorders, and incomplete questionnaire data. Data on demographics (age, height, weight, body mass index (BMI), smoking and drinking status, marital status). clinical diagnosis, main symptoms, and treatments were collected via interviews and medical records. Serum creatinine (Scr), estimated Glomerular Filtration Rate (eGFR), urea nitrogen (BuN), Albumin-to-Creatinine Ratio (ACR), and blood uric acid (BUA) were obtained from the latest laboratory results. The eGFR was calculated using the CKD-EPI (Chronic Kidney Disease Epidemiology Collaboration) equation, standardized by isotope-dilution mass spectrometry, excluding the CKD-EPI 2021 race-free eGFR formula. Due to incomplete cystatin C data and its applicability in the Chinese population^10^. The study followed the principles of the Declaration of Helsinki and was received ethical approval from the Ethical Committee of Guangdong Provincial Hospital of Chinese Medicine. Informed consent was obtained from all patients involved in the study.

### Questionnaire

The sexual dysfunction of patients was evaluated by questionnaires called 5-item version of the International Index of Erectile Function (IIEF-5) and Premature Ejaculation Diagnostic Tool (PEDT) in Chinese version. IIEF-5 consists of five items focusing on erectile function and degree of satisfaction in sexual intercourse. Additional analyses of the questionnaire help to distinguish the presence and severity levels of ED: normal ED (22–25 points), mild ED (12–21 points), moderate ED (8–11 points), and severe ED (1–7 points)^11^. PEDT was used for the assessment of PE. PEDT scores ≥ 11 indicates PE diagnosis, 9 or 10 refers to probable PE, while ≤ 8 indicates nonexsitence of PE^12^. At the same time, PE was considered the patient has an Intravaginal Ejaculation Latency Time (IELT) of less than 3 minutes and is not sexually satisfied.

Furthermore, psychological disorder is often associated with renal failure and sexual disability. Depression is an independent risk factor in male CKD patients with sexual dysfunction^13^. Thus, the Patient Health Quentionnnaire-9 (PHQ-9) and General Anxiety Disorder-7 (GAD-7) were applied to all patients to evaluate psychological states. PHQ-9 is a nine-item questionnaire designed to screen for depression. Scores varied from 5, 10, 15, to 20 represent mild, moderate, moderately severe, and severe depression, respectively^14,15^. GAD-7 is a screening tool for detecting anxiety. The cut-points of ≥ 5, ≥ 10 and ≥ 15 stand for mild, moderate and severe anxiety, respactively^16,17^.

### Statistical analysis

Data were entered into Excel and verified by three investigators. Statistical analyses were performed using R version 3.5.2 (R Foundation). Patient characteristics and sexual function data, stratified by CKD stages, were analyzed using Student's t test (t-test) and were presented as mean ± SD for continuous variables. Pearson's correlation analysis tested relationships between variables, While count data were presented as median or percentage with group comparisons made using the Kruskal-Wallis H test. *P* value < 0.05 was considered statistically significant. Correlation heatmaps and Spearman correlation analysis evaluated the association between sexual function and factors such as renal function indicators, psychological state, and medications. Simple linear regression identified potential risk factors for sexual function (*P* < 0.2), leading to multivariable linear regression analysis (via stepwise method) to determine the significant predictors. This process assessed the impact of baseline characteristics on sexual function indicators. (IIEF-5 scores, PEDT scores and IELT).

### Flow chart

**Figure 1:**
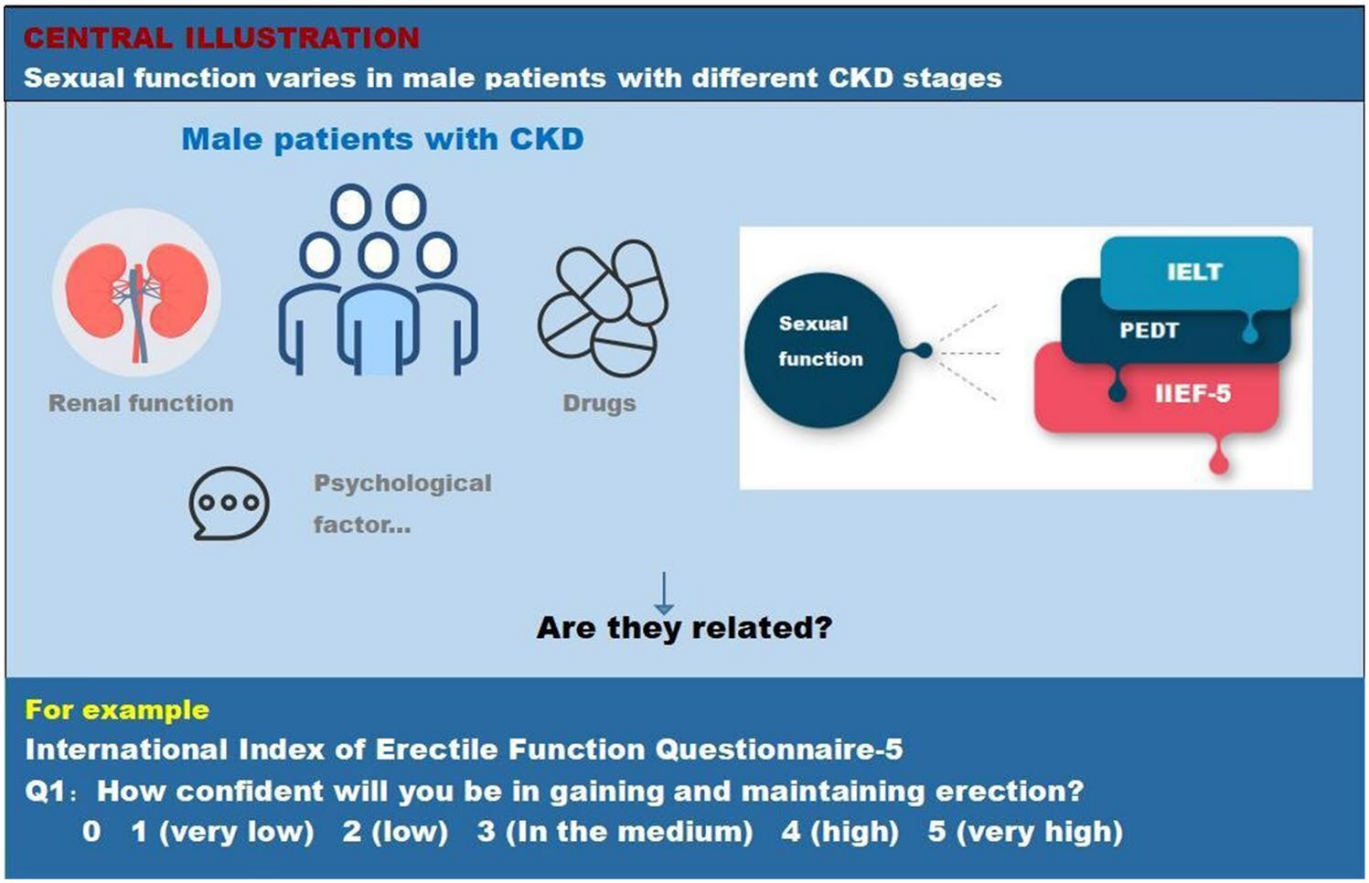
Central Illustration.

**Figure 2:**
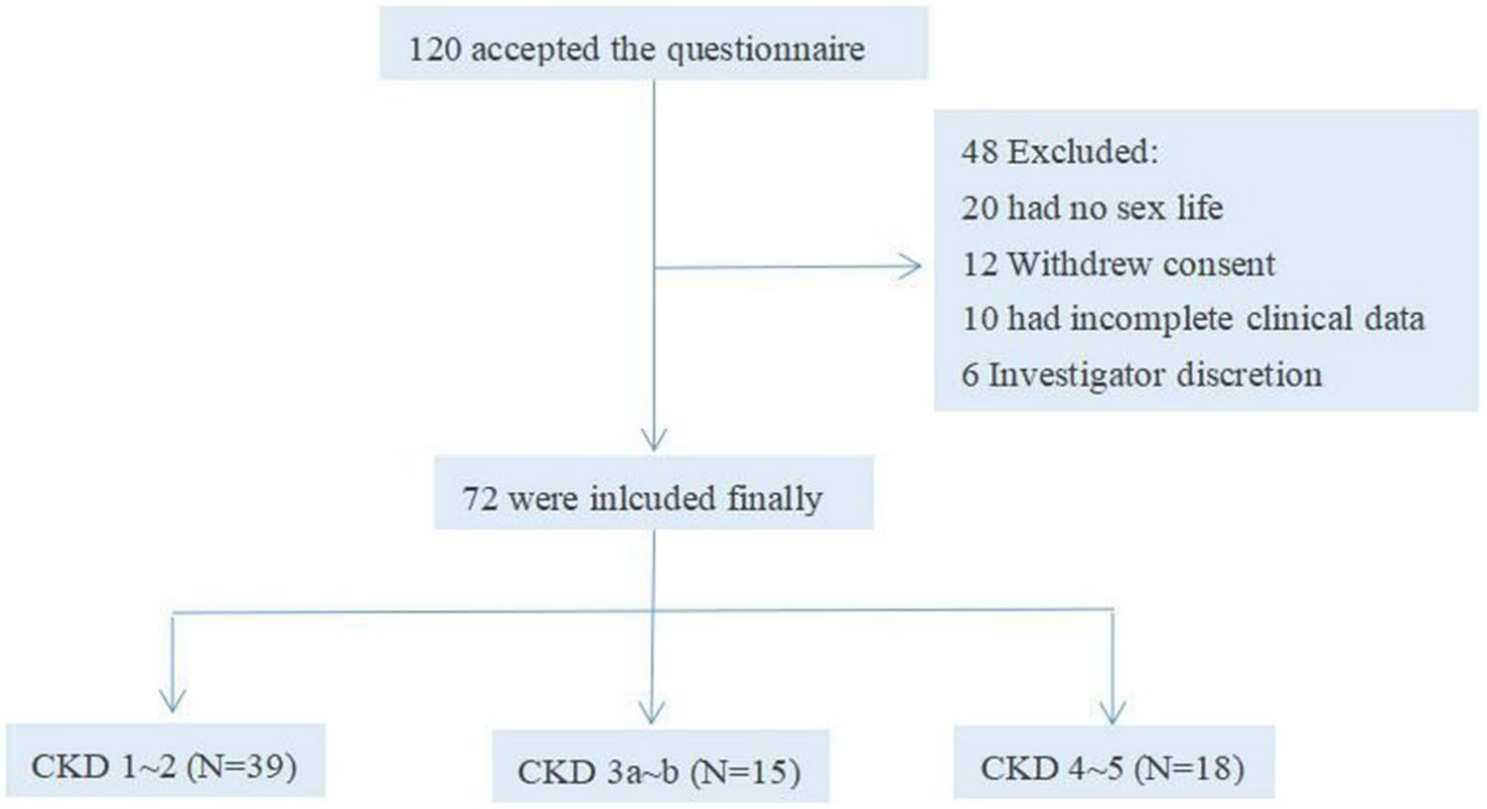
Clinical trial flowchart.

A total of 120 male CKD patients consented to participate and completed the questionnaire. Of these, 48 patients were excluded for various reasons: 20 patients had not been sexually active in the past year, 12 withdrew their informed consent, 10 had incomplete clinical data, and 6 were excluded at the discretion of the researcher. Consequently, 72 patients were included in the final analysis. These patients were categorized according to their CKD stages. The recruitment process and categorization are illustrated in Figs. 1 and 2.

### Informed consent

Informed consent was obtained from all subjects involved in the study.

## Results

### Patient baseline characteristics

Table 1 presents the baseline characteristics of the 72 enrolled patients, categorized by their CKD stages. There were 39 patients in CKD stages 1–2, 15 in stages 3a–3b, and 18 in stages 4–5. The average age at inclusion was 43.94 ± 8.93 years. Patients in CKD stages 1–2 averaged 40.90 ± 8.10 years, those in stages 3a–3b were 50.20 ± 6.54 years old, and patients in stages 4–5 were 45.33 ± 9.65 years old. The age difference across CKD stages was statistically significant (*P* = 0.001), with patients in stages 1–2 being younger. Marital status, height, weight, BMI, and drinking status showed no significant differences across the groups. The overall mean Scr was 246.72 μmol/L, displaying a large variation and thus a non-normal distribution. The average eGFR was 62.22 ± 36.24 mL/min/1.73 m^2^. ACR values varied widely, with an average of 1256.50 mg/L. Specially, the ACR of CKD was 648.42 mg/L in stages 1–2, 874.61 mg/L in stage 3, and 2892.25 mg/L in stages 4–5, with these differences being statistically significant (*P* < 0.001), indicating a correlation between higher Scr and ACR levels in patients. BUA levels did not significant differ among the groups. The prevalence of associated conditions such as hypertension (HTN), diabetes mellitus (DM), and benign prostatic hyperplasia varied across CKD stages. Regarding medication usage, differences were observed in the administration of calcium channel blockers (CCB), β-blockers, and ISA across different stages of CKD. For psychological aspects, the overall depression scale score averaged 7.12 ± 5.76, with 6.08 ± 4.94 in stages 1–2, 5.07 ± 5.26 in stage 3, and 11.11 ± 6.19 in stages 4–5. The increased depression score in higher CKD stages was statistically significant (*P* = 0.002), suggesting that the higher the CKD stage, the more depressed the patient was. Anxiety scale scores showd no significant variance across groups. Detailed data are provided in Table 1.

**Table 1:** Baseline patient characteristics by stages of CKD.

	Overall (N = 72)	CKD 1–2 (N = 39)	CKD 3a–3b (N = 15)	CKD 4–5 (N = 18)	*P* value
Demographics					
Age, y	43.94 ± 8.93	40.90 ± 8.10	50.20 ± 6.54	45.33 ± 9.65	0.001
Marital status (Married, %)	63 (87.50)	32 (82.10)	15 (100.00)	16 (88.90)	0.199
Weight, kg	68.86 ± 10.76	68.15 ± 12.12	70.37 ± 9.76	69.17 ± 8.59	0.791
Height, cm	168.71 ± 5.37	168.44 ± 5.80	168.60 ± 4.90	169.39 ± 5.00	0.825
BMI, kg/m^2^	24.18 ± 3.59	23.98 ± 3.98	24.75 ± 3.25	24.14 ± 3.08	0.780
Smoking status (%)					
Current smoker	12 (16.70)	5 (12.90)	3 (20.00)	4 (22.20)	0.032
Ex-smoker	10 (13.90)	1 (2.60)	5 (33.30)	4 (22.20)	
Never	50 (69.40)	33 (84.60)	7 (46.70)	10 (55.60)	
Drinking status (%)					
Current drinker	13 (18.10)	7 (17.90)	4 (26.70)	2 (11.10)	0.270
Ex-drinker	10 (13.90)	3 (7.70)	2 (13.30)	5 (27.80)	
Never	49 (68.10)	29 (74.40)	9 (60.00)	11 (61.10)	
Renal function					
Scr, μmol/L	**246.72 ± 311.03**	92.84 ± 18.06	143.61 ± 22.91	666.06 ± 391.12	< 0.001
eGFR, mL/min/1.73 m^2^	62.22 ± 36.24	89.96 ± 19.11	49.97 ± 8.53	12.32 ± 8.66	< 0.001
ACR, mg/L	1256.50	648.42	874.61	2892.25	< 0.001
BuN, mmol/L	10.11 ± 8.65	5.21 ± 1.20	8.29 ± 5.17	22.22 ± 8.60	< 0.001
BUA, μmol/L	410.63 ± 136.26	387.27 ± 102.33	440.77 ± 105.83	436.12 ± 205.36	0.289
Comorbidities (%)					
Hypertension	44 (61.10)	18 (46.20)	9 (60.00)	17 (94.40)	0.002
Diabetes mellitus	11 (15.30)	2 (5.10)	2 (13.30)	7 (38.90)	0.004
Chronic prostatitis	4 (5.60)	4 (10.30)	0 (0.00)	0 (0.00)	0.167
Benign Prostate Hyperplasia	4 (5.60)	0 (0.00)	2 (13.30)	2 (11.10)	0.079
Medication regimen (%)					
ACEI/ARB	25 (34.70)	16 (41.00)	6 (40.00)	3 (16.70)	0.178
CCB	18 (25.00)	1 (2.60)	5 (33.30)	12 (66.70)	< 0.001
β-blockers	9 (12.50)	1 (2.60)	0 (0.00)	8 (44.40)	< 0.001
ISA	24 (33.30)	18 (46.20)	3 (20.00)	3 (16.70)	0.042
ULT	28 (38.90)	12 (30.80)	5 (33.30)	11 (61.10)	0.081
SGLT2	2 (2.80)	1 (2.60)	1 (6.70)	0 (0.00)	0.506
Other hypoglycemic drugs	5 (6.90)	2 (5.10)	1 (6.70)	2 (11.10)	0.71
Psychological factor					
Depression	7.12 ± 5.76	6.08 ± 4.94	**5.07 ± 5.26**	11.11 ± 6.19	0.002
Anxious	3.24 ± 3.18	3.08 ± 3.24	2.73 ± 2.58	4.00 ± 3.51	0.476

Significant values are in bold.

Values are mean ± SD, n (%), or median (IQR).

CKD: chronic kidney disease; BMI: body mass index; Scr: serum creatinine; eGFR: estimated glomerular filtration rate; ACR: albumin-to-creatinine ratio; BuN: urea nitrogen; BUA: blood uric acid; AECI/ARB: Angiotensin converting enzyme inhibitor, Angiotonin II receptor blocker; CCB: calcium channel blocker; β-blockers: Beta blockers; ISA: immunosuppressant; ULT: Urate-Lowering Therapy; SGLT2: sodium-dependent glucose transporters 2.

### Results of correlation heatmap of sexual function, renal function, demographic data, diagnostic information, psychological factors and drug factors

**Figure 3:**
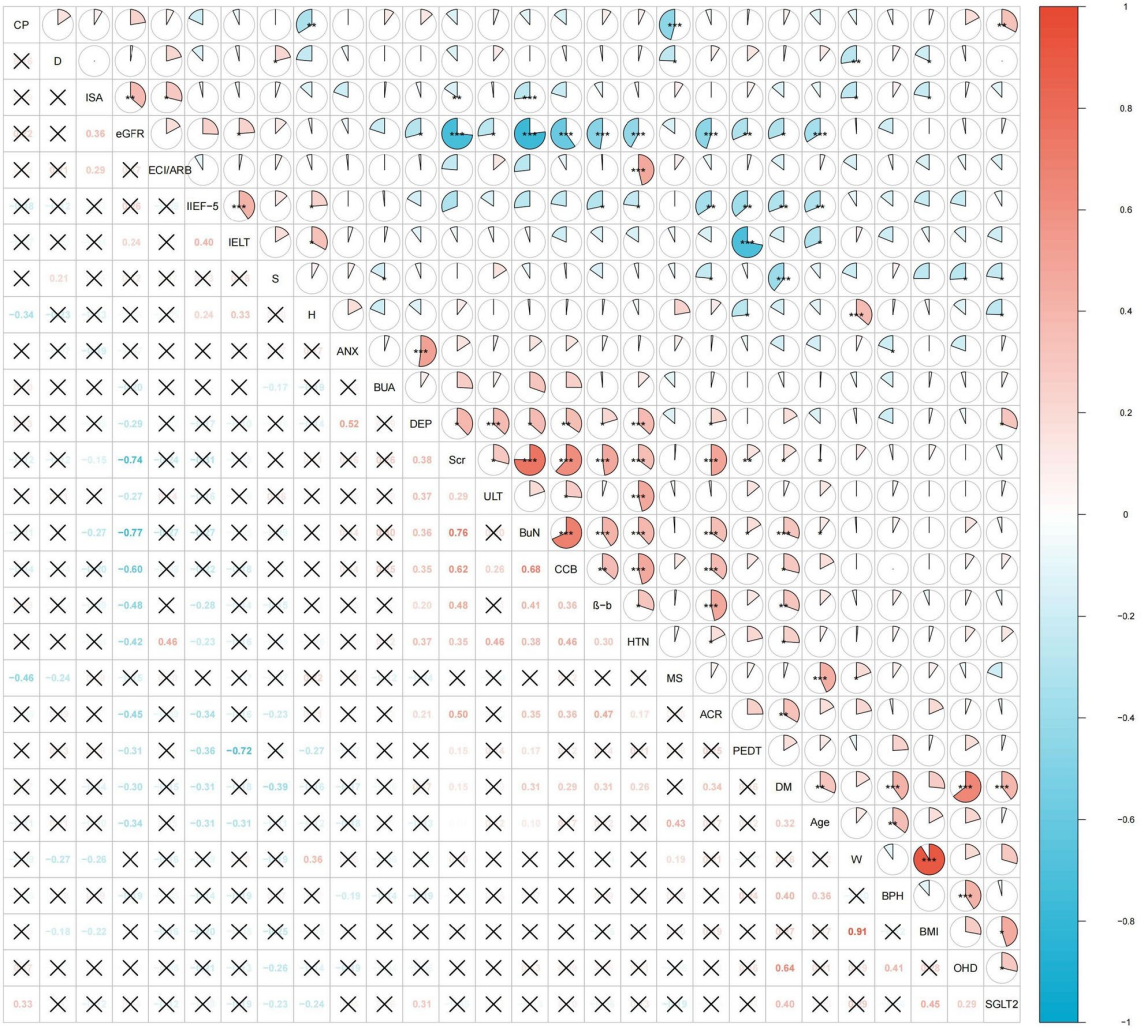
Correlation Heatmap. Marital status: MS; Weight: W; Height: H; Chronic prostatitis: CP; Hypertension: HTN; Diabetes mellitus: DM; Benign Prostate Hyperplasia: BPH; Drinking status: D; Smoking status: S; BMI: body mass index; Scr: serum creatinine; eGFR: estimated glomerular filtration rate; ACR: albumin-to-creatinine ratio; BuN: urea nitrogen; BUA: blood uric acid; AECI/ARB: Angiotensin converting enzyme inhibitor, Angiotonin II receptor blocker; CCB: calcium channel blocker; β-b: β-blockers; ISA: immunosuppressant; ULT: Urate-Lowering Therapy; SGLT2: sodium-dependent glucose transporters 2; Depression: DEP; Anxious: ANX. Correlation Heatmap was generated like the following step: Firstly, the data was imported into the R 4.3.1 version to calculated correlation coefficient. Secondly, the corrplot 0.92 package was downloaded for correlation heatmap drawing.

Figure 3 shows a correlation heatmap where asterisks (*) indicate the statistical significance of P values, with more asterisks denoting higher significance. A larger the fan area in the heatmap's upper right circular figure and a higher numerical correlation coefficient in the lower left corner suggest stronger correlatios. A correlation coefficient above zero denotes a positive correlation, whereas one below zero indicates a negative correlation. As illustrated in Fig. 3, sexual function indices such as IIEF-5 scores, IELT, and PEDT scores were associated with various influencing factors. IIEF-5 scores exhibited a weak positive correlation with height and a weak negative correlation with β-blockers and HTN with strong negative correlations with ACR, DM, and age. Notably, IIEF-5 scores and ACR had a correlation coefficient of − 0.34 (**), suggesting an inverse relationship where higher ACR levels were associated with lower IIEF-5 scores, indicating more severe ED. IELT had positive correlations with height and negative correlations with age, showing a weak positive correlations with eGFR, indicated by a correlation coefficient of 0.24 (*). PEDT scores showed very weak positive correlations with BuN and Scr, and a weak negative correlation with height, but a strong negative correlation with eGFR with a correlation coefficient of − 0.31 (**), implying that lower eGFR levels correspond to higher PEDT scores and more severe PE.

### Results of simple linear regression analysis assessing the risk factors of sexual dysfunction

Initially, simple linear regression included all variables for univariate screening, with categorical variables undergoing dummy variable transformation. Table 2 reveals that significance for demographic characteristics (age, height, smoking status), renal function (Scr, eGFR, ACR, BuN), comorbidities (HTN, DM, CP), medication regimen (CCB, β-blockers, ULT, Other hypoglycemic drugs), and depression were *P* < 0.2 when examining their association with IIEF-5 scores. Consequently, these variables were incorporated into the multifactorial model. Similarly, for IELT simple linear regression, *P* values for demographic characteristics (age, height, smoking status), renal function (eGFR, ACR), comorbidities (HTN, DM, BPH), and medication regimen (CCB, SGLT2) were < 0.2, warranting their inclusion in the multivariable linear regression analysis. For PEDT scores analysis, variables such as demographic characteristics (height, smoking status), renal function (eGFR, ACR, BuN), comorbidities (HTN, DM, BPH), and medication regimen (Other hypoglycemic drugs) also had *P* values < 0.2 and were thus carried forward for further.

**Table 2:** Results of simple linear regression analysis assessing the risk factors of sexual dysfunction.

Dependent variable	IIEF-5 Scores (*p*)	IELT (*p*)	PEDT scores (*p*)
Demographics			
Age	0.008*	0.008*	0.309
Marital status (married)	0.940	0.545	0.524
Weight	0.457	0.608	0.536
Height	0.045*	0.004*	0.024*
Smoking status			
Ex-smoker	0.242	0.022*	0.610
Never	0.170*	0.045*	0.458
Drinking status			
Ex-drinker	0.202	0.252	0.208
Never	0.698	0.877	0.007*
Renal function			
Scr	0.008*	0.530	0.036*
eGFR	0.030*	0.046*	0.165*
ACR	0.003*	0.184*	0.998
BuN	0.024*	0.663	0.073*
BUA	0.882	0.783	0.172*
Comorbidities			
Hypertension	0.053*	0.187*	0.876
Diabetes	0.008*	0.131*	0.043*
Chronic prostatitis	0.129*	0.577	0.744
Benign Prostate Hyperplasia	0.232	0.112*	0.301
Medication regimen			
ACEI/ARB	0.471	0.797	
CCB	0.060*	0.121*	0.243
β-blockers	0.016*	0.244	0.505
ISA	0.760	0.724	0.238
ULT	0.192*	0.640	0.738
SGLT2	0.505	0.117*	0.168*
Other hypoglycemic drugs	0.071*	0.282	
Psychological factor			
Depression	0.153*	0.386	0.947
Anxious	0.980	0.678	0.579

Simple linear regression was used to analyse the association between baseline characteristics and sexual function indicators (including IIEF-5 scores, PEDT scores and IELT). Statistical significance was set at *P* < 0.2

### Results of multivariable linear regression analysis (via stepwise method)

Utilizing stepwise multiple linear regression, IELT was found to be unrelated to renal function indices (*P* > 0.05) and was instead associated with demographic characteristics (age, height). Three regression models were developed to examine the correlation between IIEF-5 scores and significant influencing factors (*P* < 0.05). The third model, with an adjusted R 0.210 was deemed the most effective, explaining 21% of the variation in IIEF-5 scores. Age, height, and ACR were significant predictors in this model, with age (β = − 0.225, *P* = 0.041), height (β = 0.129, *P* = 0.024), and ACR (β = − 0.332, *P* = 0.003) all impacting IIEF-5 scores. ACR had the most substantial negative effect on IIEF-5 scores (β = − 0.332), with a slope of − 0.001, indicating a one-point decrease in IIEF-5 scores for each 1 g increase in ACR, holding other variables constant. More details can be found in Table 3. The correlation of PEDT scores with various factors was assessed through two regression models, all found to be statistically significant (*P* < 0.05) with modle 2 showing the largest R^2^ (0.153), indcating that height and eGFR accounted for 15.3% of the variance in PEDT scores. In model 2, height (β = − 0.278, *P* = 0.013), and eGFR (β = − 0.325, *P* = 0.004) were statistically predictors. eGFR, with the largest negative effect (β = − 0.325), suggesting a decrease of 0.042 in PEDT scores for every 1 mL/min/1.73 m^2^ increase in eGFR. The detailed results of these analyses are provided in Table 4.

**Table 3:** Results of multivariable linear regression analysis (via stepwise method) assessing the influencing factors of IIEF-5 scores.

Model	Unstandardized coefficient	Standardized coefficient	*P*	Collinearity statistics		R	Adjusted R^2^
	B	β		Tolerance	VIF		
Model 1: Independent Variable: ACR						0.118	0.106
ACR	− 0.001	− 0.344	0.003	1.000	1.000		
Model 2: Independent Variable: ACR, Height						0.195	0.172
ACR	− 0.001	− 0.375	0.001	0.988	1.012		
Height	0.336	0.279	0.013	0.988	1.012		
Model 3: Independent Variable: ACR, Height, Age						0.243	0.210
**ACR**	**− 0.001**	**− 0.332**	**0.003**	**0.951**	**1.051**		
Height	0.299	0.129	0.024	0.968	1.033		
Age	− 0.163	− 0.225	0.041	0.951	1.051		

We set ACR, height and age as independent variables and IIEF-5 scores as dependent variables to make multivariable linear regression analysis (via stepwise method). Model 3 was the best (R^2^ = 0.21).

Significant values are in bold.

**Table 4:** Results of multivariable linear regression analysis (via stepwise method) assessing the influencing factors of PEDT scores.

Model	Unstandardized coefficient	Standardized coefficient	*P*	Collinearity statistics		R	Adjusted R^2^
	B	β		Tolerance	VIF		
Model 1: Independent Variable: eGFR						0.099	0.086
eGFR	− 0.041	− 0.315	0.007	1.000	1.000		
Model 2: Independent Variable: eGFR, Height						0.177	0.153
**eGFR**	**− 0.042**	**− 0.325**	**0.004**	**0.999**	**1.001**		
Height	− 0.244	− 0.278	0.013	0.999	1.001		

We set eGFR and height as independent variables and PEDT scores as dependent variables to make multivariable linear regression analysis (via stepwise method). Model 2 was the best (R^2^ = 0.153).

Significant values are in bold.

## Discussion

This study reveals a high prevalence of sexual dysfunction among male CKD patients, that intensifies with renal function decline. Sexual dysfunction in these patients correlates with vascular lesions, neuropathy, psychological aspects, and medication use. The crucial role of the kidneys in circulatory balance and oxygen delivery to tissues, including sexual organs, underscores the impact of renal function on sexual health. Male sexual dysfunction refers to the inability to obtain satisfaction in sexual behavior and have a normal sexual life, which seriously affects the quality of life. The most common sexual dysfunctions in men are ED and PE^1^. Scr, eGFR and ACR are effective markers in assessment of renal function. With the progression of CKD, Scr and ACR gradually increased, eGFR decreased, which may affect sexual function in male patients. We also found that ACR was negatively correlated with IIEF-5 scores, and eGFR was negatively correlated with PEDT scores. As such, sexual function appears to worsen as renal function declines.

### ED in Male Patients with CKD

**Figure 4:**
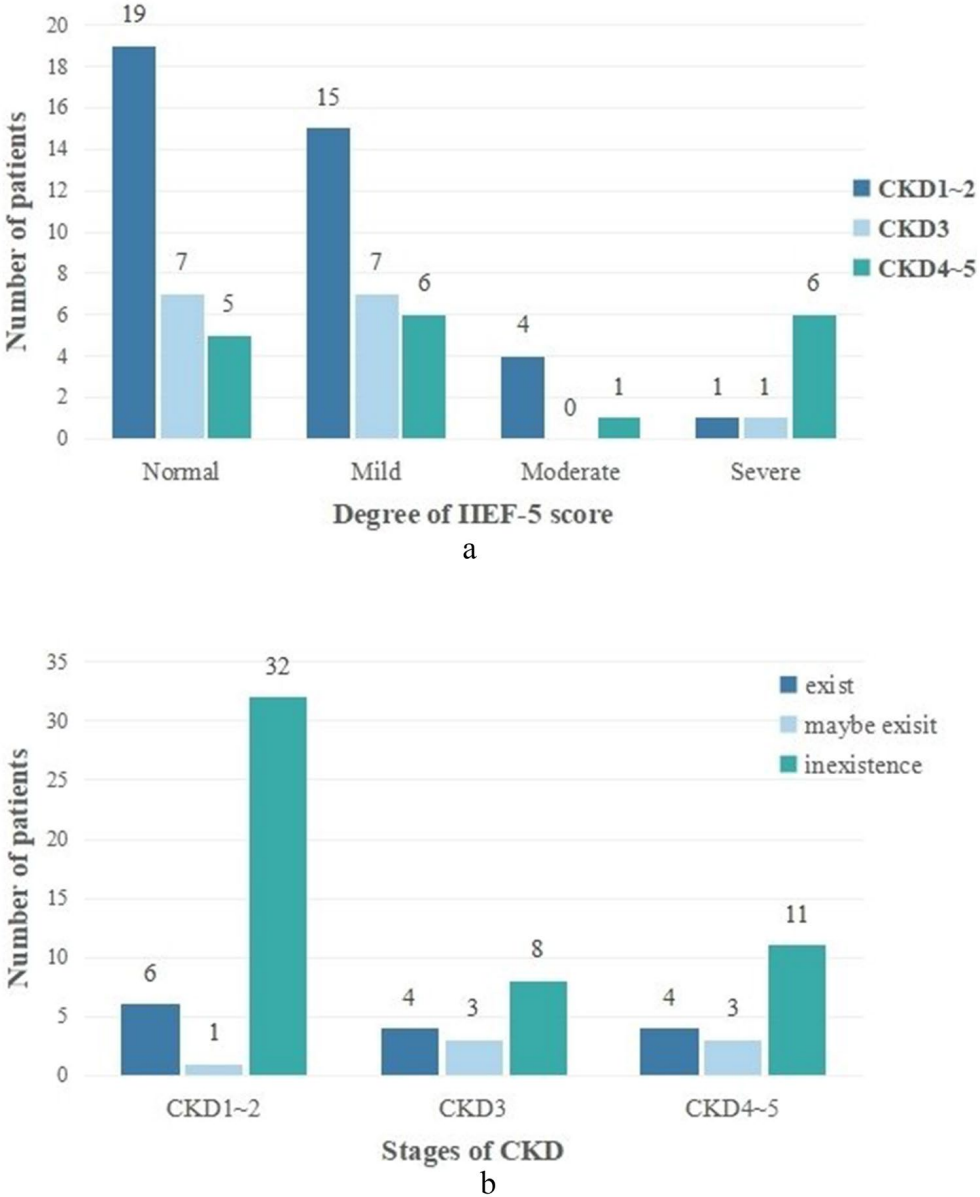
(**a**) Degree of IIEF-5 score in patents with CKD. (**b**) Degree of PEDT score in patients with CKD.

Among the participants, 41 males with CKD exhibited ED, translating to a prevalence rate of 56.9%, as detailed in Fig. 4a. Previous studies have reported, ED prevalence ranging from 9 to 84% in CKD patients undergoing conservative treatment^2,4^. Damiano et al. found that 76% of males with CKD experienced ED based on a meta-analysis of 34 studies encompassing 5986 subjects^6^. The relatively lower prevalence of ED in our study might be ascribed to the limited sample size and potential cultural influences.

Research by Momoh et al.^18^ and Neuzillet et al.^13^ identified hypertension and diabetes as contributing factors to ED in CKD patients, likely due to the resultant vascular and neurological damage. The interplay between the vascular and nervous systems is critical for normal erectile function. Hypertension a primary cardiovascular condition is known to precipitate atherosclerosis and endothelial dysfunction in penile vasculature. CKD Patients frequently suffer from hypertension, with prevalence rate reaching 70–95%^8^. Furthermore, CKD expedites vascular aging, with cardiovascular disease being a major cause of morbidity and mortality^9^. ED, manifesting ahead of cardiovascular disease, serves as a significant predictor for the latter^11^. Thus, early detection of ED might facilitate CKD prognosis and management. Additionally, both sympathetic and parasympathetic nervous systems play essential roles in achieving and maintaining a penile erection. Uremia and diabetic nephropathy often lead to polyneuropathy, impairing erectile function^12^. A comprehensive study involving 2,869 men indicated that diabetes triples the risk of ED^15^, suggesting a strong relationship between vascular and neurological impairments and ED in CKD patients. However, our study did not establish a definitive correlation between hypertension, diabetes and sexual function, possibly due to sample size limitation.

Contrary to our findings, Serkan et al. identified depression as an independent factor influencing sexual dysfunction in males with CKD^14^. Conversely, our results align with Furqan et al., who observed no significant link between depression and ED in male CKD patients at stage 5^16^. Theofilou et al. even reported a negative correlation between ED and depression in CKD patients, further complicating the picture^17^. Despite the varied perspectives on the relationship between ED and depression in CKD patients, depression appears to have a detrimental impact on sexual dysfunction in this group. The burden of CKD and its associated complications can be overwhelming, often leading to depression, due to concerns such as Unemployment, financial strain, and anxiety over future treatments, This depressive state can cause a lack of sexual desire and a reduction in the frequency of sexual activity^14^, which in turn, can negatively influence the psychological well-being of patients^5^.

While our study did not establish a direct association between medications and IIED-5 scores, PEDT scores, or IELT, the broader impact of polypharmacy, particularly with anti-hypertensive medications, on sexual dysfunction is recognized. Research has shown that β-blockers are inversely related to IIEF-5 scores, indicating a detrimental effect on sexual function^19,20^. Karavitakis et al. reported that sexual dysfunction was more prevalent in patients treated with β-blockers compared to a placebo group, with an incidence of 21.6%^21^. The negative impact of β-blockers on sexual health is attributed to their suppression of the sympathetic nervous system and reduction in testoterone levels^22^. Conversely, consistent with our findings, other study have indicated that CCB do not significantly influence sexual function^19,23,24^.

Endocrine disturbances, particularly in the hypothalamic-pituitary-gonadal (HPG) axis, are intrinsically linked to ED in CKD patients, although endocrine assessments were not part of our study^25^. CKD-related changes disrupt the secretion of gonadotropin-releasing hormone (GnRH) and consequently affect gonadotropin production. The progression of CKD, marked by decreased eGFR, often leads to increased levels of luteinizing hormone (LH) and follicle-stimulating hormone (FSH), impacting testosterone synthesis^26^. Additionally, uremic in CKD patients can alter the negative feedback of LH and FSH, further diminishing testosterone levels^27^. Furthermore, elevated prolactin levels, common in CKD, exacerbate this issue, as hyperprolactinemia reduces gonadotropin secretion leading to ED and hypogonadism^28,29^.

### PE in male CKD patients

The definition of PE lacks universal consensus. Employing the evidence-based medical definition of PE by the ISSM expert group, we utilized the PEDT scale and IELT to evaluate PE, offering both subjective and objective perspectives on its incidence. Our findings showed that 29.2% of non-dialysis CKD male patients experienced PE, as shown in Fig. 4b. Notably, research on PE among CKD patients, particularly at early stages, is sparse. A study in the Brazilian Amazon reported a 36.7% occurrence of PE among hemodialysis CKD patients^30^. Elbardisi et al. found an 88.24% prevalence of PE in patients with advanced CKD^31^. Variations in PE prevalence may be attributed to differing study criteria and methodologies.

Our study suggest a potential positive correlation between renal function deterioration and PE. Supporting this, Serkan et al. observed that stage 5 CKD patients exhibited higher PE scores compared to those in stages 3 and 4^14^. While the precise causes of PE remain to be identified, psychological and neurological factors are increasingly recognized as significant contributors^32^. CKD patients frequently face psychological stress, which may influence the onset of PE. Moreover, conditions such as diabetic nephropathy, associated with CKD, likely contribute to PE. Additionally, PE might be linked to abnormalities in serotonergic neurotransmission^33^, and studies in animal models indicate that renal dysfunction impacts serotonin metabolism^34^. Thus, PE in CKD patients may stem from both psychological and neurological changes.

In contrast, our study failed to confirm the correlation between hypertension and PE, diverging from some prior findings^33,35,36^. This discrepancy leads to the hypothesis that antihypertensive medications might induce PE in CKD patients, warranting further investigation.

### Limitations

Despite yielding important insights, our study has some limitations. Our investigation was small-numbered and single-center designed, which may affect the statistical robustness of the findings. Moreover, the reliance on self-assessment questionnaires introduces potential biases. Future research will enlarge the sample size and utilize more objective assessment methods. Despite these constraints, our research provides important knowledge on PE in predialysis CKD patients.

## Conclusion

Sexual dysfunction, notably prevalent in CKD-affected males, is multifactorial. We advocate for increased clinical attention to sexual dysfunction in male CKD patients and call for further investigation into its underlying mechanisms.

## Data Availability

The datasets used and analyzed during the current study are available from the corresponding author on reasonable request.
